# Coronary Artery Anomalies: A Short Case Series and Current Review

**DOI:** 10.7759/cureus.38732

**Published:** 2023-05-08

**Authors:** Md Mashiul Alam, Tasniem Tasha, Ammy S Ghosh, Farjana Nasrin

**Affiliations:** 1 Internal Medicine, Bridgeport Hospital/Yale University, Bridgeport, USA; 2 Internal Medicine, Fralin Biomedical Research Institute, Virginia Tech, Blacksburg, USA; 3 Hematology-Oncology, University of Michigan, Ann Arbor, USA; 4 Oncology, Howard University Hospital, Washington, DC, USA

**Keywords:** intrinsic coronary artery anomaly, double right coronary artery, coronary artery fistula, coronary artery anomalous origin, coronary artery

## Abstract

Coronary artery anomalies (CAAs) are rare congenital cardiovascular defects that can present in various ways depending on the origin, course, and termination of the abnormal coronary artery fistula. It is sometimes detected incidentally during procedures such as coronary angiography or autopsies. While adults with this condition are often asymptomatic, some may experience angina, congestive heart failure, myocardial infarction, cardiomyopathy, ventricular aneurysms, or sudden cardiac death (SCD). In fact, it is the second leading cause of SCD among young athletes and requires more studies to handle such patients efficiently. To illustrate the many possible manifestations of this unusual diagnosis, we present a series of five cases. We have also reviewed the different varieties of this rare congenital anomaly and discussed the latest diagnostic tests and treatment plans.

## Introduction

Coronary artery anomalies (CAAs) are rare congenital defects involving the epicardial coronary arteries. The reported incidence of such anomaly in the literature ranges from 1% to 6% of the population undergoing a coronary angiography procedure and 0.3% of all autopsies [1-3]. Hypertrophic cardiomyopathy (HCM) is the most common cause of sudden cardiac death (SCD) among athletes, and it is the second leading cause of sudden cardiac death in young competitive athletes [4]. The clinical presentations of CAAs can vary, ranging from asymptomatic presentation to chest pain, dyspnea, and dysrhythmia leading to SCD, depending on the course of coronary arteries [5].

It is critical to have precise morphological criteria for describing normal coronary arteries. A variety of morphological aspects of the coronary arteries, such as the number of ostia, their location, course, and termination, can be identified. We must also distinguish between normal coronary anatomy variants and proper coronary anomalies [5]. When anatomical characteristics of the coronary arteries are found in more than 1% of the general population, they should be regarded as variations rather than congenital defects [1,6].

CAAs can be classified based on coronary artery origin, course or intrinsic coronary anatomy, or anomalies of coronary termination [7]. Although invasive coronary angiography has been used to diagnose coronary anomalies, coronary computed tomography angiography (CCTA) and cardiac magnetic resonance (CMR) have emerged as reliable noninvasive options for diagnosis and long-term follow-up [8]. Although it is a well-recognized clinical entity in cardiovascular medicine, it lacks well-described guidelines about managing different types of this anomalous condition [9-11]. It is imperative to have a more detailed understanding of coronary anomalies and their optimum management.

In our case series, we have presented five different types of CAA: anomalous origin, course, and termination. All our patients are adults and had coronary angiograms due to ischemic symptoms, and the cases are collected from the adult cardiology department. At the end of our case series, we have included a detailed literature review in the discussion section. We hope it will help us to understand how to classify, diagnose, and treat such cardiac anomalies irrespective of age and presentation.

## Case presentation

Case 1

Common Origin of Right Coronary Artery (RCA) and Left Coronary Artery (LCA) From the Right Coronary Sinus

A 46-year-old woman who was previously normotensive and nondiabetic presented with exertional chest discomfort for two months. The pain, centrally located with no radiation, lessens after rest. She has no history of palpitation, shortness of breath, dizziness, or orthopnea.

**Figure 1:**
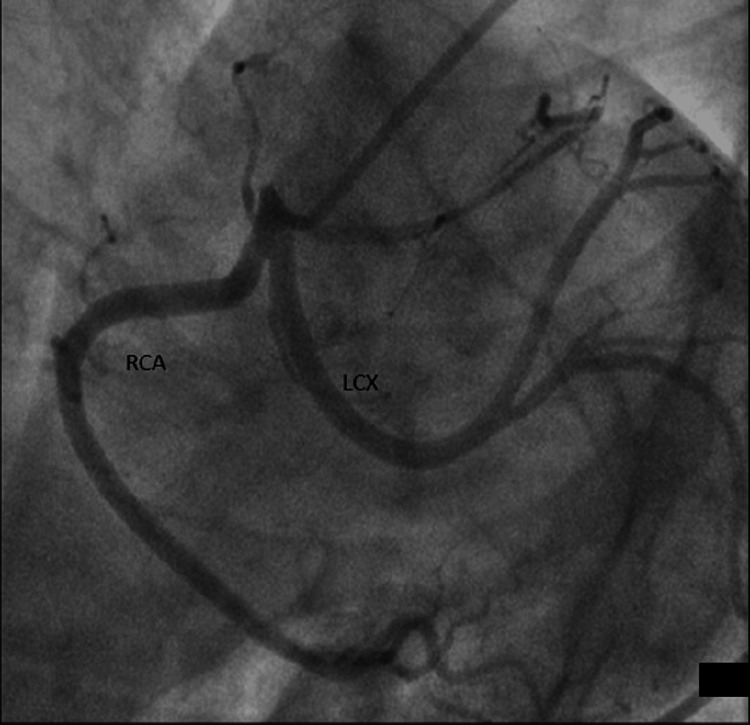
LAO cranial view showing the clear origin of RCA and LCX from this view LAO: Left anterior oblique; RCA: Right coronary artery; LCX: Left circumflex artery.

**Figure 2:**
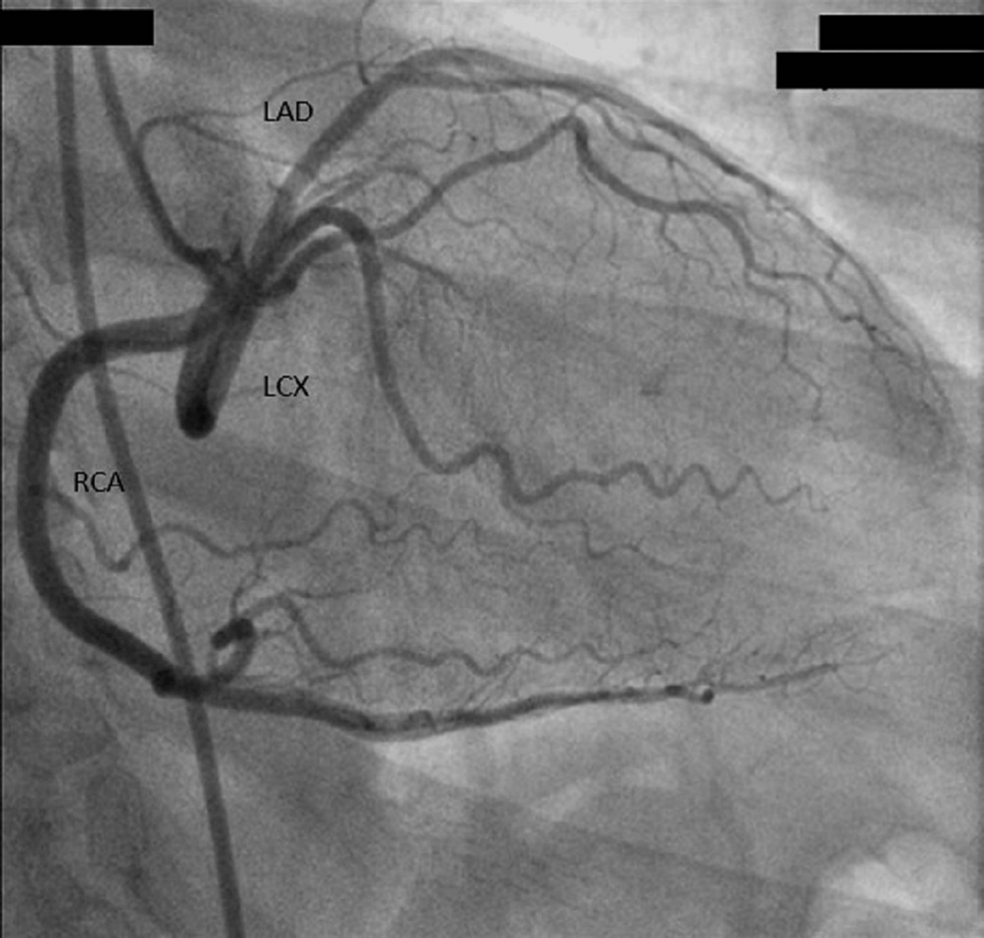
All coronary arteries from the same right coronary sinus RCA: Right coronary artery; LCX: Left circumflex artery; LAD: Left anterior descending artery.

On examination, her pulse was 80 beats per min and regular, her blood pressure was 120/80 mmHg, and she had no anemia or peripheral edema. Her BMI was 25 kg/m². Physical examination revealed normal findings. Her electrocardiogram (ECG) shows nonspecific ST changes, and echocardiography reveals no regional wall motion abnormalities with good left ventricle (LV) systolic function and wall thickness. During the coronary angiogram procedure, we could not engage the left Judkins catheter, and the root aortogram shows no artery arising from the left coronary sinus. So, the right Judkins catheter was introduced, and the right coronary sinus was engaged. It showed the common origin of RCA and LCA from the right aortic sinus of the Valsalva. All vessels had good caliber and TIMI III flow (Figures 1, 2).

Case 2

Right Coronary Artery (RCA) From the Left Coronary Sinus

A 52-year-old farmer with bronchial asthma, an ex-smoker, was referred by a urologist before renal stone surgery as the patient was complaining about burning chest discomfort after mild exertion for one month, which subsided after taking rest. This discomfort radiated to the neck only and was associated with upper abdominal pain. There was no relation between this discomfort with meal intake. There was no significant drug history. He had a low socioeconomic status.

On examination, he was ill-appearing and had mild pallor. There were no cyanosis, clubbing, and koilonychia. The jugular venous pressure (JVP) was not elevated, and there was no edema. His blood pressure was 150/100 mmHg, and his pulse was 80 beats per min and regular. The respiratory rate was 16 breaths per min, and the temperature was normal. Precordium examination revealed regular first and second heart sounds. There was no gallop or murmur. Auscultation of the lungs reveals a few crepitations and Ronchi throughout the lung field. Another systemic examination was within normal limits.

**Figure 3:**
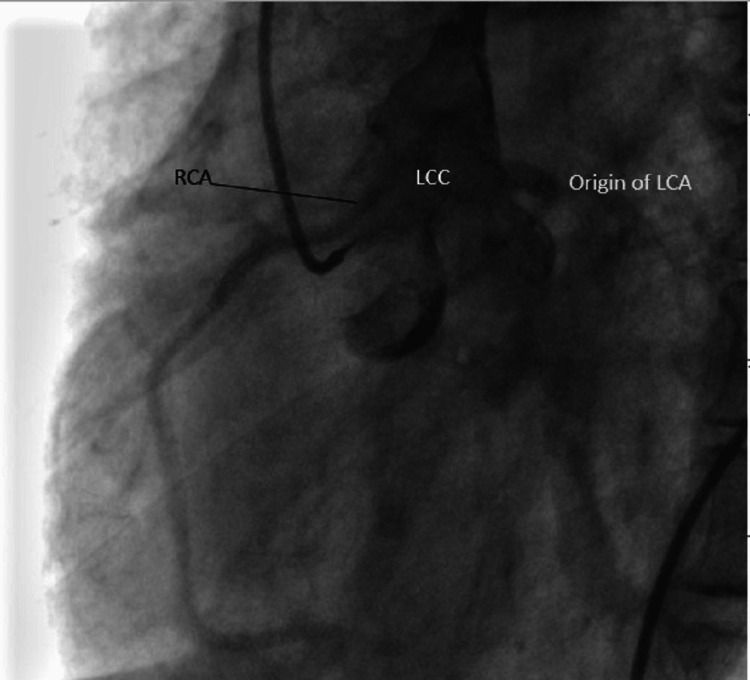
RCA from LCC RCA: Right coronary artery; LCC: Left coronary cusp; LCA: Left main coronary artery.

**Figure 4:**
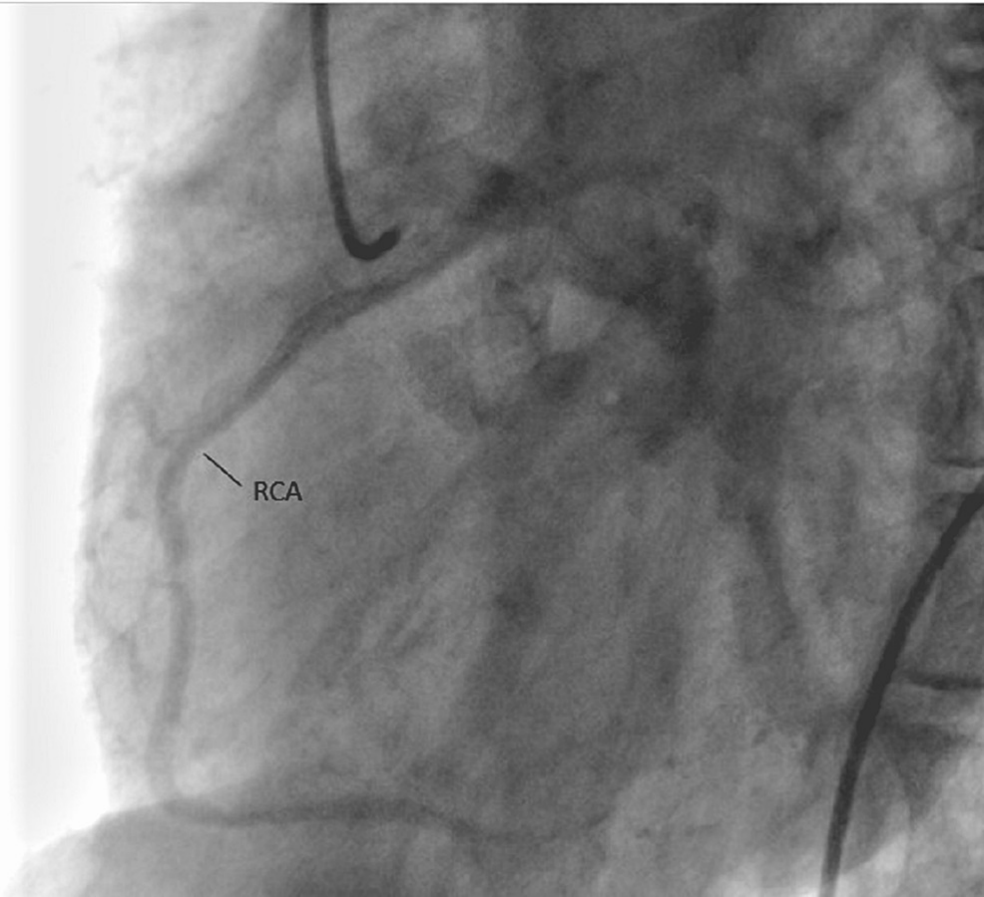
RCA course after origin from LCC RCA: Right coronary artery; LCC: Left coronary cusp.

Electrocardiogram (ECG) was within normal limits apart from nonspecific ST changes. Chest radiography (CXR) showed bilateral inflammatory lesions. Subsequent echocardiography showed no regional wall motion abnormalities with good systolic function (EF: 64%). But the exercise tolerance test (ETT) was positive for provocative myocardial ischemia. A coronary angiogram showed an 80% stenotic lesion in LAD at the level of the D2 branch with an anomalous origin of the RCA from the left coronary cusp (LCC). We could not engage a separate right coronary sinus with the right Judkin catheter (Figures 3, 4).

Case 3

Left Circumflex (LCX) From Right Coronary Cusp (RCC)

A 55-year-old businessman, ex-smoker, with known diabetes mellitus for eight years and hypertension for four years was admitted for coronary angiogram through the outpatient department due to left shoulder pain, which was compressive, aggravated after exertion, and relieved after rest. He denied any chest pain or discomfort. He had been on regular medication for his hypertension and diabetes mellitus. An ETT was done by modified Bruce protocol four years ago due to similar shoulder pain, and it was negative for provocative myocardial ischemia. The patient has been on anti-ischemic medication since then. His shoulder pain again came back following the discontinuation of the anti-ischemic drug. None of his family members had known of coronary artery disease. His general physical examination and cardiovascular examination revealed normal findings.

**Figure 5:**
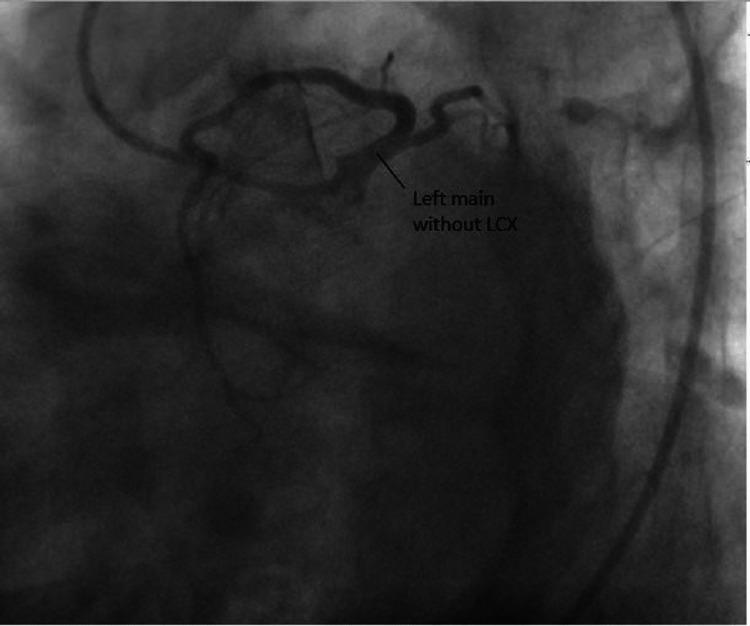
LM and LAD from the LCC LM: Left main artery; LAD: Left anterior descending artery; LCC: Left coronary cusp; LCX: Left circumflex artery.

**Figure 6:**
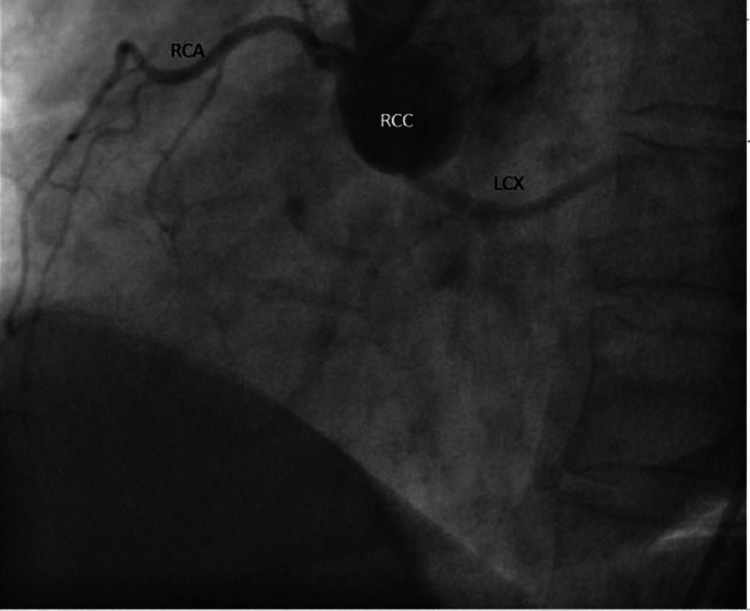
LCX from the RCC RCC: Right coronary cusp; RCA: Right coronary artery; LCX: Left circumflex artery.

**Figure 7:**
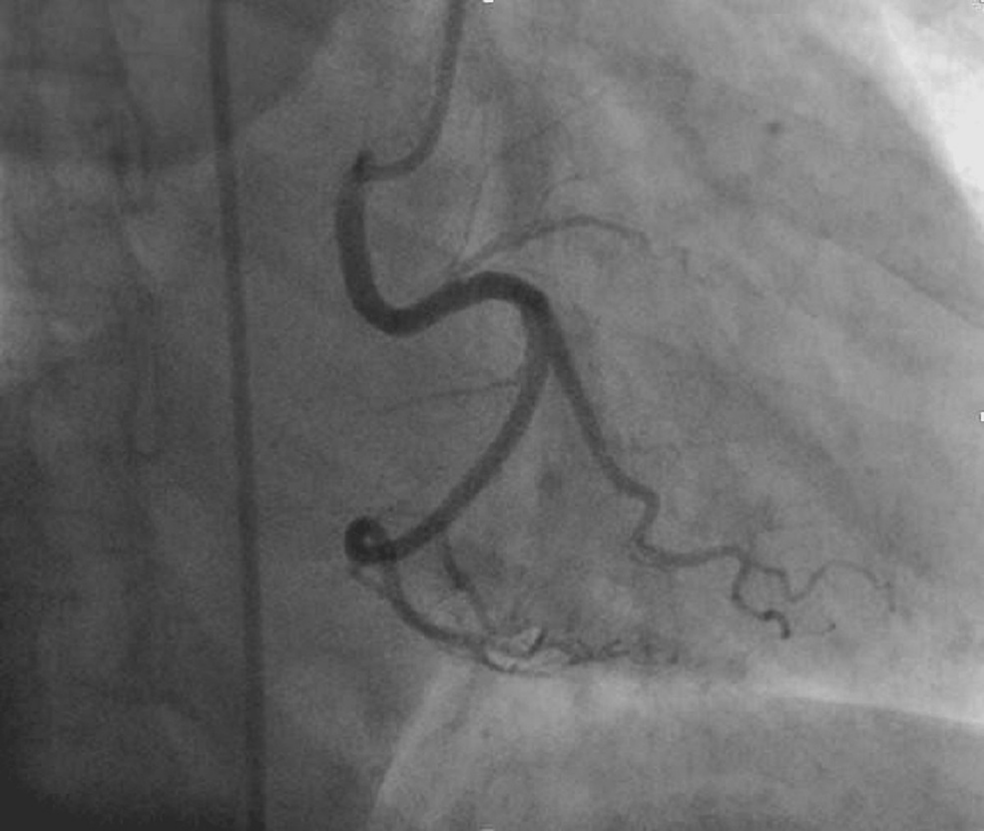
LCX from the separate ostium of RCC RCC: Right coronary cusp; LCX: Left circumflex artery.

Electrocardiogram (ECG) was almost normal except for flat “T” wave inversion in lead augmented vector left (aVL). Echocardiography showed trivial mitral regurgitation with grade-I diastolic dysfunction but good systolic function (EF: 59%) and no regional wall motion abnormality. After proper preparation, a coronary angiogram was done through the transfemoral route, which revealed the origin of the left circumflex (LCX) from the RCC but normal epicardial coronary arteries. His RCA was non-dominant (Figures 5-7).

Case 4

Split/Double Right Coronary Artery (RCA)

A 45-year-old businessman, currently a smoker, normotensive, and nondiabetic, presented with central chest discomfort with radiation to the left arm on mild exertion for the last month. He has a significant family history of coronary artery disease. Physical examination revealed normal findings.

**Figure 8:**
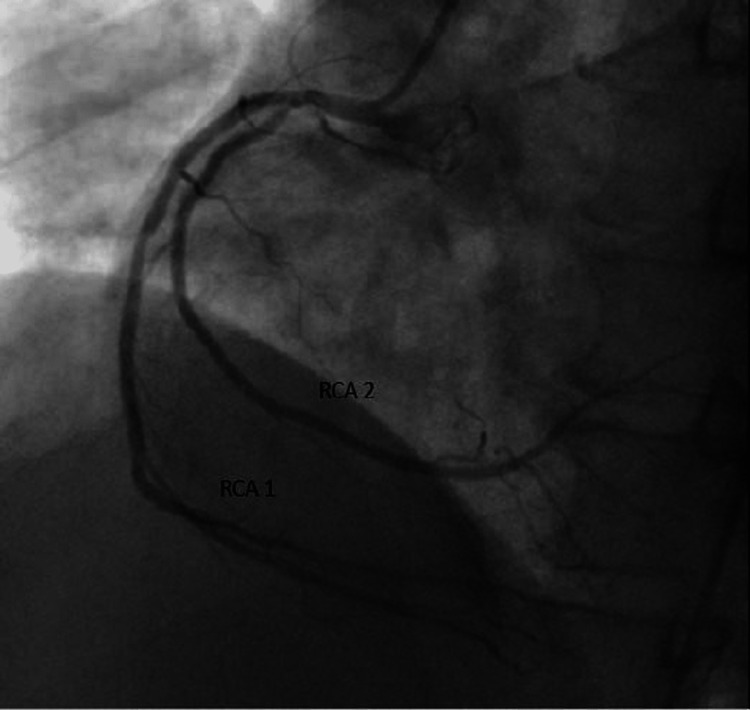
Two separate vessels from the right coronary cusp (RCA 1 and RCA 2) RCA 1: Right coronary artery 1; RCA 2: Right coronary artery 2.

**Figure 9:**
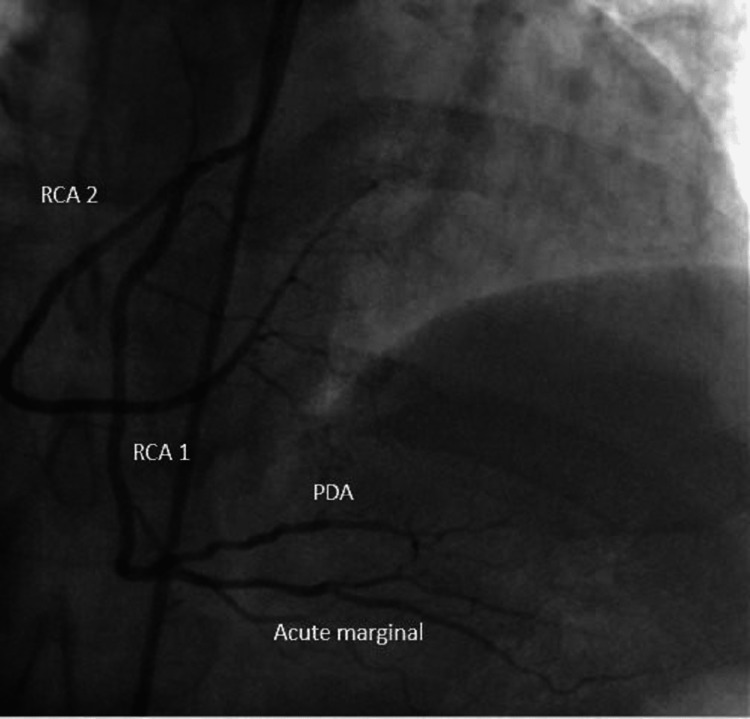
RCA1 with all branches of RCA with a separate RCA2 supplying LV LV: Left ventricle; PDA: Patent ductus arteriosus; RCA: Right coronary artery; RCA 1: Right coronary artery 1; RCA 2: Right coronary artery 2.

ECG showed nonspecific ST changes. The ejection fraction was average with normal wall motion in the resting echocardiogram. As the patient had chest pain on mild exertion, a newer onset ETT was not done, and the patient was referred for a coronary angiogram (CAG). The CAG showed left main short with more than 50% stenosis, LCX small caliber vessel with 50%-60% eccentric lesion after the second obtuse marginal branch (OM2), and little plaque before OM3-long segment stenosis of proximal left anterior descending artery (LAD) from the origin. A separate branch of RCA from the proximal segment has a similar caliber supplying the LV. It has a little plaque at the origin. The patient was sent for coronary artery bypass as the left main is involved (Figures 8, 9).

Case 5

Coronary Artery Fistula

A 57-year-old lady with hypertension presented with chronic chest discomfort that was more on exertion. Initially, the patient was treated with antihypertensive and anti-ischemic medication. Even after blood pressure control and regular intake of anti-ischemic medications, her chest pain persisted.

**Figure 10:**
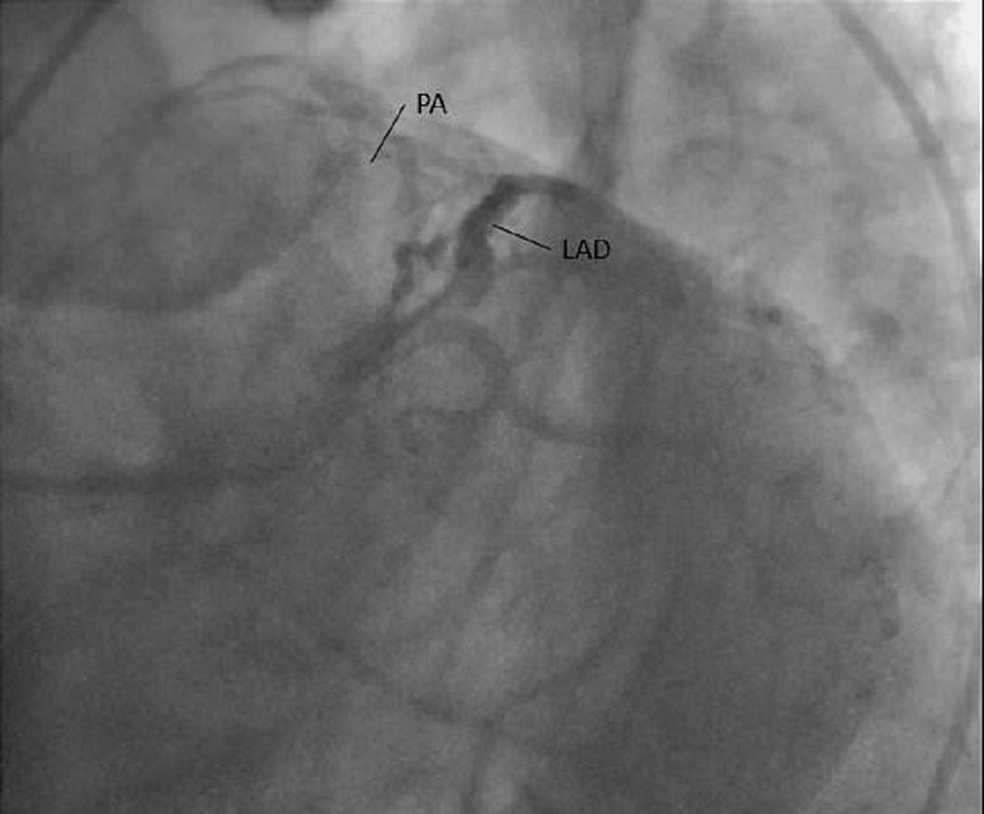
LAD and PA before the visible sprouting of blood from the fistula vessel LAD: Left anterior descending artery; PA: Pulmonary artery.

**Figure 11:**
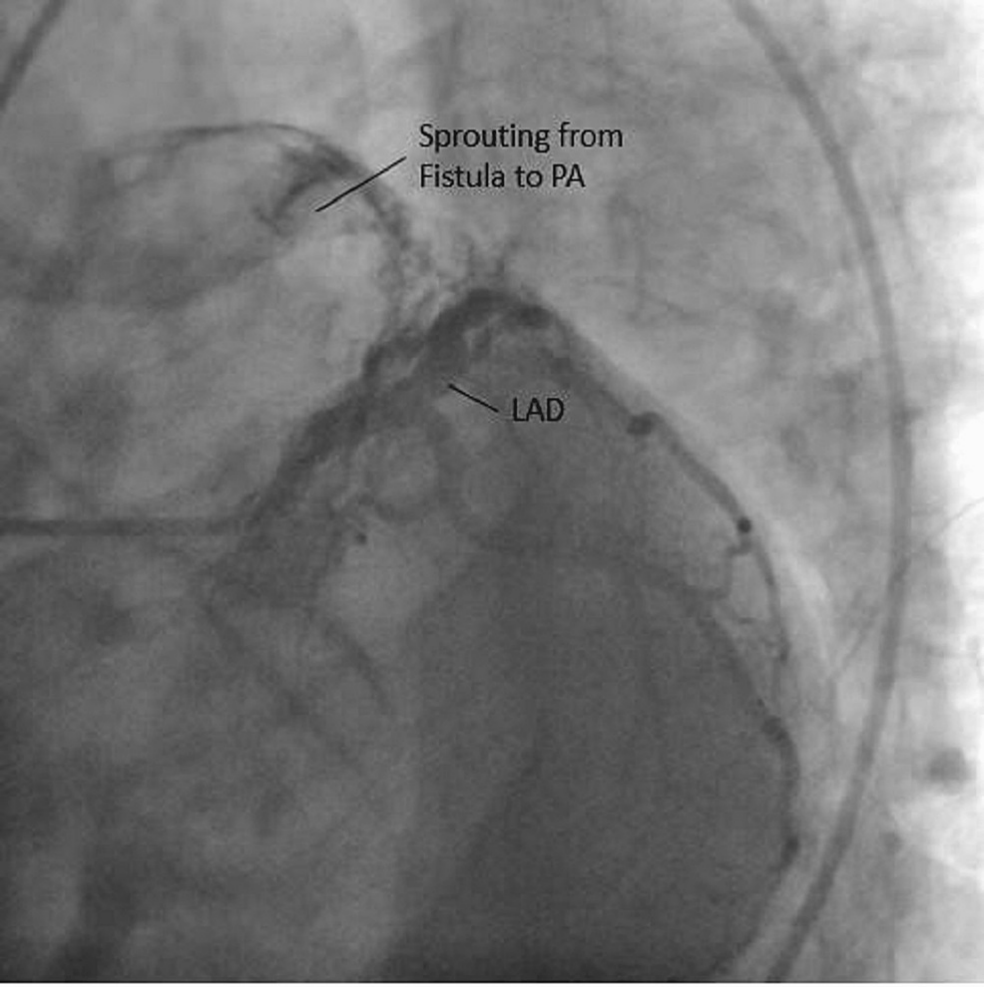
Visible sprouting from the fistula vessel from LAD to PA LAD: Left anterior descending artery; PA: Pulmonary artery.

Eventually, the patient was evaluated by a cardiologist. ECG showed nonspecific ST changes in the anterior leads. The exercise treadmill test had equivocal findings. The patient underwent a CAG as her chest pain did not improve with medical treatment. The CAG showed the typical origin of all vessels with Thrombolysis in Myocardial Infarction grade 3 (TIMI III) flow, but there was a small vessel from LAD sprouting to the pulmonary artery (PA). The patient's chest pain on exertion was explained by this fistula causing the shunting of blood from LAD to PA, causing demand ischemia during exertion. She was referred to a cardiac surgeon for further management (Figures 10, 11).

## Discussion

CAAs encompass a diverse group of abnormalities in coronary anatomy, ranging from silent bystanders to sudden cardiac death [4]. As mentioned earlier, the incidence of CAA is between 1% and 6% [1-3]; another study [8] found it in 0.78%-1.3% of the population using invasive coronary angiography. In contrast, the incidence is.99% to 5.8% in CCTA studies due to the higher sensitivity of CCTA and its broader inclusion criteria [12].

Most CAAs do not have any clinical presentation and are often found incidentally during the evaluation of another cardiac disease. However, some anomalies can be intermittent, acute, sustained, or chronic from early infancy, resulting in angina, congestive heart failure, myocardial infarction, cardiomyopathy, ventricular aneurysms, or SCD. Later, it was notably observed during or immediately after physical activity [13]. After hypertrophic cardiomyopathy, which accounted for 36% of SCDs, anomalous coronary arteries accounted for 17% (119 of 1049) of SCDs according to data analysis from an extensive US National Registry of Competitive Athletes spanning 27 years (1980-2006) [4].

Anomalous origin

Anomalies of origin feature the anomalous aortic origin of the coronary artery from the opposite or non-coronary sinus or origin from the main PA or its branches. Variations in the aortic origin of the coronary arteries are also associated with other congenital heart diseases such as tetralogy of Fallot, transposition of the great arteries, double outlet right ventricle, univentricular hearts, and truncus arteriosus [14].

Even though rare coronary arteries may arise from various other structures, including the LV and systemic arteries, such as the internal mammary artery or great aortic vessels [15], the "high" origin of a coronary artery from its respective sinus, above the level of the sinotubular junction, is prevalent in the RCA anomalous origin and is frequently associated with a bicuspid aortic valve [15]. Reduced diastolic coronary artery blood flow may result from a high origin of ostia [16]. Preoperative knowledge of high origin can facilitate successful coronary catheterization or intraoperative aortic clamping [16].

Variations in the number, shape, and position of the ostia or origins of the coronary arteries may occur in otherwise normal individuals. The majority of these variations appear clinically insignificant [17]. Anomalous coronary origin from the opposite sinus can occur on the left or right [18]. The LCA can emerge from the opposite or RCC, and similarly, the RCA may originate from the opposite or left coronary cusp [18].

The most common anomaly depending on the anomalous origin of the sinus is the RCA from the left sinus of Valsalva (8.9% of all CAAs; overall incidence: 0.08%) [5,19]. LCX from the right sinus of the Valsalva has almost similar incidence [19]. Only 1.7% of coronary artery anomalous cases of the LCA (LAD and LCx) arise from the right sinus of Valsalva (overall incidence: 0.016%) [19].

In 0.25%-0.5% of all congenital cardiac anomalies, the LCA can have an aberrant origin from the PA, called ALCAPA (anomalous origin of the LCA from the PA) [20]. In that case, collateral vessels develop in response to chronic myocardial hypoperfusion from poorly oxygenated PA blood. Reduced left ventricular myocardial perfusion results from a reversal of blood flow from the anomalous coronary artery to the PA when the right to left coronary collateralization is significant [21,22]. With recurrent myocardial infarctions, ischemic cardiomyopathy, and eventually valvular pathology, this steal phenomenon is linked to higher morbidity and death [21,22].

SCD is also linked to anomalous coronary artery origin, irrespective of age. After adjusting the presence of atherosclerotic coronary disease and other concurrent diseases, it has been associated with increased mortality in people of all ages [23]. However, SCD is most commonly seen among people with an abnormal course between the great vessels [24,25].

Anomalous course

The anomalous course of coronary arteries could be intramural (myocardial bridging), intra-arterial, pre-pulmonic, sub-pulmonic, retro-aortic, and retro-cardiac [26]. The coronary artery course between the aorta and PA to the LV may result in vessel compression, myocardial ischemia, and sudden death in adults and teenagers [24,25]. The intramural course and acute takeoff angle may predispose to obstruction to blood flow. The length of the intramural course was observed to correspond with symptoms in one study [27].

Although rare, complications commonly occur in patients with anomalous coronary arteries during or immediately after exercise; even without prior symptoms, sudden death may occur. It has been postulated that movement causes aortic root and pulmonary trunk expansion, which, in addition to external coronary artery compression, may exacerbate the pre-existing angulation of the coronary artery takeoff, reducing the luminal diameter in the coronary artery's proximal portion [24]. Furthermore, while symptoms such as exertional dyspnea and chest pain may be the earliest clinical signs of CAA, it has been demonstrated that CAA-related sudden cardiac death (SCD) can develop in 50% of previously asymptomatic persons [28]. Therefore, it is essential to carefully assess a subject's eligibility for sports competition while considering both the coronary anatomy and the existence of inducible ischemia.

The RCA's origin from the left sinus of Valsalva or the LAD from the right sinus of Valsalva can cause myocardial ischemia and sudden death. The origin of the LCX from the RCA, on the other hand, is widely thought to have little clinical relevance due to its posterior course to the LV. However, it is conceivable that vascular compression will develop.

It has been proposed that anomalous coronary arteries' abnormal origin and course of anomalous coronary arteries are more prone to atherosclerosis, but roughly 15% of such individuals might develop myocardial ischemia without atherosclerosis. The average age of symptomatic presentation was 35 years, with the oldest patient being 83 years old [29]. Notably, in our case series, the patients became symptomatic by the age of 45-57.

Normal coronary arteries run over the heart's epicardial surface and might occasionally contain an intramyocardial segment. A "myocardial bridge" is the muscle that covers the intramyocardial section of an epicardial coronary artery and is characterized by systolic compression of the tunneled segment. Myocardial bridging likely involves the proximal and mid-segment of the LAD, although it can also happen in the circumflex, diagonals, and RCA [30]. The reported prevalence of myocardial bridging among coronary angiography patients is 0.5%-2.5%, whereas it was found in 15%-85% of cases in a pathological analysis [30]. Patients with hypertrophic cardiomyopathy (HCM) frequently have the myocardial bridge, with an incidence of up to 30% [31]. The majority of myocardial bridging cases are not clinically significant. However, extensive bridging of the main coronary arteries can result in stress cardiomyopathy, myocardial ischemia, coronary thrombosis, and myocardial infarction [32]. Though coronary bridging is not associated with accelerated atherosclerosis, plaque formation immediately proximal to the bridged part is more likely [33].

The double RCA is one of the rarest coronary anomalies [34]. It can be found in isolation or associated with other congenital heart diseases. The incidence ranges from 0.01% to 0.07% [34]. A review article by Angelini [5] claimed that split RCA is the most common CAA (1.23%). Still, their definition of split RCA differed as they included other entities apart from a double vessel originating from the same RCC and supplying the right side of the heart.

A double RCA can arise from a single ostium and split into two branches after a variable short distance from the proximal trunk, or it appears from separate ostia in the right sinus of Valsalva. Atherosclerotic coronary disease is highly prevalent in patients with a double RCA originating from a single ostium. A double RCA may be accidentally discovered during coronary angiography or cardiac operation and at risk for unanticipated consequences of atherosclerotic coronary artery disease. Patients with a double RCA should be under continuous supervision for atherosclerotic change, regardless of whether or not they get intervention [35].

Anomalies of termination

Anomalous termination of the coronary artery is mainly known as coronary artery fistula. Coronary fistulae are defined as an abnormal connection between the terminal portion of the coronary artery or its branches and a vascular space with low pressure, like the cardiac chamber, PA, coronary sinus, or superior vena cava [6,36]. A coronary artery fistula was identified in 0.9% of computed tomography angiography (CTA) patients compared to 0.05%-0.25% of traditional coronary angiography patients [37]. Coronary fistula can be congenital or acquired. The persistence of intramyocardial trabecular sinusoids is the cause of congenital fistula, whereas acquired fistula can cause trauma, with Takayasu arteritis, after cardiac surgery, post angioplasty, or following interventional device closure of ventricular septal defect [38].

The fistula can be right to left or left to right, depending on the volume of the shunt. Most commonly, it drained in the right ventricle (RV) (45%), followed by the right atrium (RA) (25%) and PA (15%) [37]. The presentation of the patients depends on the size of the fistula. Patients with small fistula are usually asymptomatic, followed by serial echocardiography [6]. Patients with large fistula can present with myocardial infarction, heart failure, arrhythmia, SCD, pulmonary hypertension, rupture, and endocarditis, usually after the age of 50 [6].

Large coronary artery fistulas need an invasive approach. But the invasive procedure for fistula closure depends on fistula morphology, course, tortuosity, and aneurysmal dilation of the afferent vessel. If the fistula is complex, a drainage site surgical ligation is done. But, if the fistula is single and non-tortuous, then percutaneous closing (e.g., coil embolization, covered stents, vascular plugs, and dedicated umbrella device) is done. All patients undergoing invasive procedures have a risk of developing infective bacterial endocarditis. Hence, antibiotic prophylaxis is recommended for all patients undergoing the process [39].

Another example of CAA is coronary artery caliber abnormalities, including narrowing due to stenosis, hypoplasia, or enlargement (ectasia or aneurysmal dilatation). Coronary artery stenosis can occur at the ostium or throughout the coronary artery. These lesions can be congenital or acquired. Congenital coronary artery stenosis causes the development of rich collaterals; therefore, anginal symptoms are not expected as acquired coronary artery stenosis. Different types of vasculitis, e.g., syphilis, Kawasaki arteritis, and Takayasu arteritis, are associated with acquired coronary artery stenosis or caliber abnormalities [40].

Diagnosis

In a patient with an anomalous coronary artery, physical examination and diagnostic studies are usually unrevealing in the absence of myocardial infarction or symptoms of ongoing ischemia [24]. SCD among competitive athletes is more concerning as almost half do not have any previous ischemic symptoms but die from SCD due to anomalous coronary artery [14].

When an anomalous coronary artery origin is suspected among children, echocardiography with Doppler flow mapping is the best initial investigation to establish the diagnosis [41]. The Doppler modality of echocardiography is used to identify the anomalous origin of coronary arteries and the intramural course of vessels [9].

With the improvement of cardiac magnetic resonance angiography (CMR) and CCTA, a reliable anatomic description of the coronary artery origins and the proximal course is possible. The American College of Cardiology (ACC) and the American Heart Association (AHA) 2018 Adult Congenital Heart Disease Management Guidelines recommend coronary angiography with catheterization, CCTA, or CMR to evaluate an anomalous coronary artery [14]. But according to the American Association of Thoracic Surgery Expert Consensus (2017), an invasive coronary artery angiogram should be performed if an anomalous artery cannot be identified by a noninvasive modality or if there is a suspicion of atherosclerotic coronary arteries [9].

After identifying an anomalous coronary artery, it should be evaluated for hemodynamic significance, particularly in athletes, using stress testing, e.g., exercise treadmill test, stress echocardiogram, and nuclear perfusion imaging [9,42,43]. However, these stress tests have low negative predictive value and cannot be reassuring even if no perfusion defect is identified; this is particularly true for exercise ECG [9]. On the other hand, nuclear perfusion imaging is more specific and sensitive than the exercise treadmill tests [14].

Treatment

Patients with anomalous coronary arteries may be present widely. They could be asymptomatic, have ischemic symptoms, or present with sudden cardiac arrest/death [9-11]. Increased myocardial oxygen demand is associated with more SCD during exercise or strenuous physical activity [44]. Therefore, activity limitation is vital in patients with anomalous coronary arteries. Anomalous origin from LCA, the intra-arterial course between the aorta and PA, obviates a patient to refrain from all sorts of competitive sports before surgical repair. Anomalous origin of RCA from the left sinus of Valsalva without cardiac ischemia or arrhythmia on a stress test and anomalous origin from PA without myocardial infarction may participate in low-intensity sports [10].

Treatment of a symptomatic CAA is almost always surgical [11] or observational if not symptomatic. Some mentions of interventional procedures must be recommended [9,45]. There are some studies to elicit the role of beta blockers in preventing SCD among patients with CAA. It may be considered in patients above 30 years old who have anomalies other than the anomalous origin of the left coronary artery [9]. In a study with 56 patients, beta-blocker was tried among patients with no SCD in five years of follow-up [46], and no study was conducted among patients under 30 years of age.

Treating patients with CCAs still has a wide gap because of its rarity of presentation [47]. Age of presentation, symptoms during the presentation, anatomy of the vessels, and provocable ischemia or ventricular arrhythmia on stress tests are considered before choosing the treatment modalities.

In 2018, a guideline from the American Heart Association/American College of Cardiology (AHA/ACC) on adult congenital heart disease [11] addressed CAAs briefly and the primary recommended surgical approach. The literature search found that the anomalous LCA from the right sinus of Valsalva or PA has a higher class of recommendation for intervention than the anomalous RCA and always recommended surgical approach irrespective of the presence of symptoms. This is because of an increased chance of SCD among patients with anomalous LCA [10,18,48]. As per expert consensus guideline from American Association for Thoracic Surgery (AATS) (2015), patients with anomalous RCA may participate in competitive sports without any need for surgical correction if there is no inducible ischemia in the imaging study [9], but the newest guideline from AHA/ACC also recommends looking for ventricular arrhythmia to take a decision [11].

Anatomy of vessels such as slit-like orifice, acute angle during takeoff, longer intramural course, the inter-arterial course between aorta and PA, and stenosis of the proximal coronary artery is associated with an increased risk of symptoms or SCD [11,44]. These factors need to be considered when making decisions regarding the management of patients.

Different hospitals also have an institutional variation of treatment approaches with their policy to treat this congenital anomaly. In one such institute, they operate on all patients aged 10-30 regardless of symptoms and have a more selective approach for patients less than or more than this age cut-off [49]. In another institute, high-risk coronary anatomy, coronary artery dominance, participation in competitive sports, or family decisions are considered before choosing a treatment option [50].

The risk of surgical procedures is low according to multiple studies [43,49,51], and choosing a surgical technique depends on various factors [9]. “Unroofing” is a general approach in patients with an intramural course of the anomalous coronary artery. Reimplantation or ostial reconstruction may be considered in a short intramural artery course patient. Coronary artery bypass grafting may be required if a patient has concomitant atherosclerotic coronary arteries, which is particularly true when the subject’s age is more than 35 years [11].

Percutaneous coronary intervention (PCI) has a limited role in CAA, particularly among young children. It may be considered in selective adult subjects with short intramural coronary artery, proximal coronary artery stenosis [44], or coronary artery fistula [40]. No large-scale study has compared the PCI and surgical approach for such procedures' long-term safety and efficacy [9].

According to AHA/ACC congenital heart disease Task Force 4 (2015), all athletes can participate in competitive sports after three months of surgical repair of “wrong sinus” origin if the patient is asymptomatic and the exercise stress test shows no evidence of provocable ischemia or cardiac arrhythmia. After surgical repair of anomalous artery origin from PA exercise, we should also look for the presence of myocardial infarction or ventricular dysfunction, which is a common complication [10]. On the other hand, patients with a history of SCD before surgical repair are at increased risk of another episode [52]. The AATS recommends not returning to competitive sports for one year after the surgical procedure [9].

As per recommendation from AATS (2017), after immediate postoperative follow-up, all patients should be in long-term follow-up with a cardiologist annually and take baby aspirin daily. For those who participate in competitive sports, they also recommend ECG and ECHO tests yearly as well as an exercise stress test every one to three years. A nuclear perfusion scan may be considered if new symptoms appear [9].

## Conclusions

CAAs are rare clinical entities. Most people are asymptomatic and present after the initial symptoms. CAA cases are increasingly found with the increasing use of invasive and noninvasive cardiovascular imaging. In our case series, we presented a few cases of anomalous course of origin, an anomalous course of termination (coronary artery fistula), and one rare case of the double RCA. It should be a differential in any young patient with ischemic symptoms or athletes with SCD. The anatomy determines the treatment of coronary artery abnormalities revealed.
